# Genetics Modulate Gray Matter Variation Beyond Disease Burden in Prodromal Huntington’s Disease

**DOI:** 10.3389/fneur.2018.00190

**Published:** 2018-03-29

**Authors:** Jingyu Liu, Jennifer Ciarochi, Vince D. Calhoun, Jane S. Paulsen, H. Jeremy Bockholt, Hans J. Johnson, Jeffrey D. Long, Dongdong Lin, Flor A. Espinoza, Maria B. Misiura, Arvind Caprihan, Jessica A. Turner, PREDICT-HD Investigators and Coordinators of the Huntington Study Group

**Affiliations:** ^1^The Mind Research Network & Lovelace Biomedical and Environmental Research Institute (LBERI), Albuquerque, NM, United States; ^2^Department of Electrical and Computer Engineering, University of New Mexico, Albuquerque, NM, United States; ^3^Department of Psychology, Georgia State University, Atlanta, GA, United States; ^4^Department of Neuroscience, Georgia State University, Atlanta, GA, United States; ^5^Department of Psychiatry, University of Iowa, Iowa City, IA, United States; ^6^Department of Neurology, University of Iowa, Iowa City, IA, United States; ^7^Department of Psychological and Brain Sciences, University of Iowa, Iowa City, IA, United States; ^8^Department of Electrical and Computer Engineering, University of Iowa, Iowa City, IA, United States; ^9^Department of Biostatistics, University of Iowa, Iowa City, IA, United States

**Keywords:** genetic modifier, gray matter, Huntington’s disease, cognition, prodromal disease progression

## Abstract

Huntington’s disease (HD) is a neurodegenerative disorder caused by an expansion mutation of the cytosine–adenine–guanine (CAG) trinucleotide in the *HTT* gene. Decline in cognitive and motor functioning during the prodromal phase has been reported, and understanding genetic influences on prodromal disease progression beyond CAG will benefit intervention therapies. From a prodromal HD cohort (*N* = 715), we extracted gray matter (GM) components through independent component analysis and tested them for associations with cognitive and motor functioning that cannot be accounted for by CAG-induced disease burden (cumulative effects of CAG expansion and age). Furthermore, we examined genetic associations (at the genomic, HD pathway, and candidate region levels) with the GM components that were related to functional decline. After accounting for disease burden, GM in a component containing cuneus, lingual, and middle occipital regions was positively associated with attention and working memory performance, and the effect size was about a tenth of that of disease burden. Prodromal participants with at least one dystonia sign also had significantly lower GM volume in a bilateral inferior parietal component than participants without dystonia, after controlling for the disease burden. Two single-nucleotide polymorphisms (SNPs: rs71358386 in *NCOR1* and rs71358386 in *ADORA2B)* in the HD pathway were significantly associated with GM volume in the cuneus component, with minor alleles being linked to reduced GM volume. Additionally, homozygous minor allele carriers of SNPs in a candidate region of ch15q13.3 had significantly higher GM volume in the inferior parietal component, and one minor allele copy was associated with a total motor score decrease of 0.14 U. Our findings depict an early genetical GM reduction in prodromal HD that occurs irrespective of disease burden and affects regions important for cognitive and motor functioning.

## Introduction

Huntington’s disease (HD) is a neurodegenerative disorder characterized by deterioration of motor, cognitive, and psychiatric functioning. Abnormal cytosine–adenine–guanine (CAG) repeat expansion (>35 repeats) in the huntingtin gene (*HTT*) causes this progressive disorder, and age of clinical diagnosis is inversely correlated with CAG expansion length (i.e., greater expansion is associated with more rapid progression) (1). Although CAG repeat number is the primary determinant of the rate of pathogenesis (explaining about 56% of the variation in onset age), overall onset time is highly variable, especially in patients with lower CAG repeat numbers (1–3). Other genetic and environmental factors likely account for additional onset variation (4–6), as illustrated by an HD pedigree study showing that approximately 40% of the variation in onset age (after accounting for CAG effects) was due to non-*HTT* genetic factors (7).

Up to a decade prior to clinical diagnosis, individuals with the abnormal CAG expansion already differ from healthy controls in brain structure as well as cognitive and motor functioning (3, 8, 9). Investigating early prodromal changes may be necessary for identifying optimal targets for disease prevention or delay (10). This is a major goal of PREDICT-HD, a multisite prodromal HD study that has characterized many features of the HD prodrome (10–12), including widespread gray matter (GM) concentration reductions [even at the earliest prodromal stage (13)], robust annual changes in putamen, caudate, and nucleus accumbens volumes, as well as metrics of motor and cognitive functioning (3), resting state functional connectivity changes (14), and subcortical brain volume variations associated with motor symptom severity, cognitive control, and verbal learning (8, 9, 15). The extensiveness of brain structural and functional changes in this population supports the suitability of brain-based phenotypes for probing early genetic effects on prodromal disease progression.

To date, several promising non-*HTT* genetic modulators, including *ADORA2A* (16, 17) and *GRIN2A-2B* (18), among others (5, 6, 19–22), have been highlighted as potential modifiers of disease onset or progression. The GeM-HD (genetic modifiers of HD) consortium conducted the largest such study, compiling genetic data from multiple projects and investigating genetic factors associated with residual variance in onset time (after controlling for CAG influence). This study identified two genomic significant loci in chromosome 15 that accelerated or delayed onset by 6.1 and 1.4 years, respectively (20). Another new study of disease progression in both prodromal and diagnosed HD patients reported an association between single-nucleotide polymorphisms (SNPs) in chromosome 5 and a reduced rate of change in motor and functional capacity scores (23). However, no study has examined genetic modulation of brain-based phenotypes during the HD prodrome.

The CAG age product (CAP), computed as age × (CAG repeat − constant), captures the cumulative effect of CAG expansion on the duration of exposure, and is a validated index of disease burden in HD (24, 25). During the prodromal phase, CAP significantly and reliably associated with brain volumetric changes and cognitive and motor decline (24), yet it cannot explain all the variation in these measures (or in clinical onset age) (3). Thus, to pinpoint non-*HTT* genetic factors that influence prodromal brain-based phenotypes, we intentionally removed CAP influence on GM variation through regression; this is analogous to the residual variance in onset time implemented in the GeM-HD study. We then identified GM networks associated with cognitive or motor decline in prodromal individuals and tested these for genetic effects.

## Materials and Methods

### Participants

715 (447 female and 268 male) PREDICT-HD prodromal individuals from 33 sites were analyzed. These participants were gene positive (with >36 CAG repeats) independent samples, and did not convert to HD during the study. All participants provided written, informed consent and were treated in accordance with protocols approved by each participating institution’s internal review board. Detailed enrollment and exclusion criteria can be found in previous publications (12). Participant demographic information is provided in Table 1. There were no differences in age, CAG repeats, or education years between males and females. 54 participants had fewer than 40 CAG repeats; even though these participants may or may not develop HD in their lifetimes, the large variability in their prodromal disease progression (which partially contributes to the uncertainty of onset) makes it more appealing to include them in the prodromal analysis.

**Table 1:** Demographic information of participants.

	715 prodromal HD	Female (*N* = 447, 62.5%)	Male (*N* = 268, 37.5%)
Age	42.55 ± 10.53 (19–83)	42.6 ± 10.5	43.5 ± 10.7
Cytosine adenine guanine repeats	42.47 ± 2.54 (37–61)	42.43 ± 2.57	42.53 ± 2.50
Education years	14.50 ± 2.61 (8–20)	14.36 ± 2.55	14.73 ± 2.69
Race (self-reported)	694 (97.06%) White	96.64% White	97.76% White
	1 American Indian	1 American Indian	1 Asians
	3 Asians	2 Asians	3 intermixed
	14 intermixed	11 intermixed	2 unknown
	7 unknown	5 unknown	
Race (genetic estimated)	97.34% Caucasian	97.09% Caucasian	97.76% Caucasian
	1 Asian	1 Asian	2 intermixed
	2 intermixed	12 Mexican/Indians	5 Mexican/Indians
	17 Mexican/Indians		

### Cognitive and Motor Functioning Assessments

Motor variables included total motor score (TMS) from the Unified Huntington’s Disease Rating Scale and the chorea, bradykinesia, oculomotor, and dystonia subdomains from the 15-item standardized motor assessment (26, 27). Many participants had low or 0 scores on the motor variables, skewing the data toward a negative exponential distribution. Cognitive variables included the Symbol Digit Modalities Test (SDMT) (27, 28), Stroop Color, Stroop Word, and Stroop Interference tests (27, 29), and Trail Making Tests A (TMTA) and B (TMTB) (27, 30, 31). Cognitive variables had approximately normal distributions. More details for each variable are available in the Supplementary Material.

Total motor score, oculomotor, bradykinesia, and chorea were highly correlated (e.g., TMS correlated with oculomotor, bradykinesia and chorea at *r* = 0.79, 0.83, and 0.70, respectively; Figure S1 in Supplementary Material). Thus, we used principal component analysis (PCA) to extract the first PC (89% of the total variance) as the representative variable for overall motor function; higher scores indicate more abnormal motor control, and the most weighted variable is TMS. Similarly, SDMT and Stroop scores were highly correlated (*r* = 0.53–0.78), and we obtained the first PC (76% of the total variance) as the representative variable for attention and working memory; higher scores indicate better performance, and the most weighted variable is Stroop Word. TMTA and TMTB were grouped and the first PC (95% of the total variance) was obtained as the representative variable for problem solving; higher scores indicate slower processing, and the most weighted variable is TMTB. For dystonia, which was not highly correlated with the other motor variables, 639 participants had scores of 0, 37 had scores of 1, 24 had scores of 2, and 5 had scores higher than 2. The low scores on dystonia are in line with the prodromal status of the participants, as dystonia is usually a sign of disease manifestation. We converted dystonia score into a binary variable representing presence or absence of dystonia signs.

### Genetic Data Preprocessing

Genomic SNP data were downloaded from dbGAP (Study Accession: phs000222.v4.p2). We removed problematic loci in accordance with PREDICT-HD quality control recommendations, and filtered SNPs for a missingness rate of 5% per sample and 5% per SNP and a minor allele frequency of 5%. Family relatedness was determined using PLINK identity-by-descent analysis, and only one member per family was included. The top 10 multidimensional scaling (MDS) factors from PLINK were used to correct for population structure. A total of 1,160,231 SNPs across the genome were investigated. In parallel, we also investigated an HD pathway derived from the Ingenuity Pathway Analysis knowledgebase and the KEGG database. The HD pathway from the two combined databases included 310 genes and 3,404 SNPs after pruning with *r*^2^ > 0.5 (see Table S1 in Supplementary Material).

### Candidate Selection

Since only prodromal patients were investigated and prodromal functional decline is more relevant to symptom onset than to disease progression [which accelerates significantly faster after onset compared to during the prodrome (23)], we selected candidate SNPs for modifying onset time; these were from the GeM-HD study, and included two regions (chr15q13.2-3: rs146353869, rs2140734; chr8: rs1037699) with significant influences on age of motor diagnosis and nominal associations with cognitive and psychiatric symptom onset (20). We tested SNPs within these regions for effects on prodromal progression. Although our data did not include these exact three SNPs, we identified seven nearby SNPs in high linkage disequilibrium (LD) with rs2140734 (*r* > 0.98 based on NIH LDlink web^1^): rs11293, rs11629793, rs8034856, rs7176569, rs35784593, rs1474380, and rs61997138. These SNPs were highly correlated in our data (*r* > 0.99), exhibiting almost identical genotype patterns. There were also three SNPs in our data with identical genotype patterns that were in high LD with rs1037699 (*r* > 0.85): rs16869295, rs11777942, and rs11778107.

### Imaging Data Processing

T1-weighted images from the earliest available MRI scans were segmented into GM, modulated, normalized to MNI space, and smoothed with an 8 mm × 8 mm × 8 mm Gaussian kernel using the statistical parametric mapping 8 software package.^2^ Images less than 80% correlated with the averaged GM were removed, and a >0.2 GM volume mask was generated to include only GM relevant voxels. Since these imaging data were collected from 50 site and scanner field strength (1.5 or 3 T) combinations, known influences of site scanner, age, sex, and disease burden on GM were removed by applying a linear regression model to each GM voxel. Site scanners were coded as 49 dummy variables, and disease burden was calculated using the formula suggested by PREDICT-HD: CAP = age × (CAG − 33.66) (24).

### Source-Based Morphometry

We then applied independent component analysis (ICA) to whole-brain GM voxels using the source-based morphometry toolbox within the GIFT software package (http://mialab.mrn.org/software/gift). ICA decomposes the brain imaging data into maximally independent GM components, often comprised of multiple brain regions, with each component/network grouping voxels that covary among subjects (32). The model can be described simply as *X* = A × S, where *X* is the measured data, *S* contains the extracted components, and *A* is the loading matrix. A participant’s loading coefficient for a given component indicates how strongly that component manifests in the participant’s imaging data [see Ref. (32–35) for details]. Fifteen GM components were estimated, as determined by the minimum description length criteria (36).

### Statistical Analyses

We first tested whether the cognitive and motor variables were significantly associated with disease burden in our prodromal sample. PCA-derived representative variables and original variables were tested one by one, separately. A regression model (cognitive or motor variable = age + sex + CAP) was used for each variable. Due to different distributions for motor versus cognitive variables, a linear regression model was used for cognitive variables, a logistic regression model was used for the converted binary dystonia variable, and a Poisson regression model was applied to the other motor variables.

Next, we tested for associations between the extracted GM components and cognitive and motor functioning variables using a regression model in which the cognitive or motor functioning variable = age + sex + GM loadings + CAP. Similarly, linear, Poisson, and logistic models were used accordingly. The GM components significantly contributing to motor or cognitive functioning after adjusting for CAP were our primary components of interest for genetic associations. For any GM component of interest, a regression model (GM loading = SNP + top 10 MDS scores) was used to test for SNP associations at the genomic, pathway and candidate levels. We also tested for associations between clinical (motor or cognitive) variables and SNPs using the following regression model: motor or cognitive variable = age + sex + CAP + SNP + top 10 MDS scores. All tests, genomic level and pathway level, were false discovery rate (FDR) corrected at *p* < 0.05 for the number of tested SNPs.

## Results

### Disease Burden and Clinical Functioning

Individual motor and cognitive variables and derived representative variables were all associated with CAP after controlling for age and sex (*p* = 0.04 for the converted binary dystonia score, *p* = 0.01 for the original dystonia score, and *p* < 1 × 10^−11^ for all other variables). Due to highly consistent results among representative variables and original individual variables, hereafter we report the results from representative measures. Results from individual variables are provided in the Supplementary Material. The total variance explained by the regression model was 19% for overall motor function (9–18% for individual variables), 18% for working memory/attention (12–21% for individual variables), and 15% for problem solving (13 and 15% for TMTA and TMTB, respectively). The pseudo *R*^2^ for dystonia was 1.3% (2% for the original dystonia score).

### GM and Clinical Functioning

**Figure 1:**
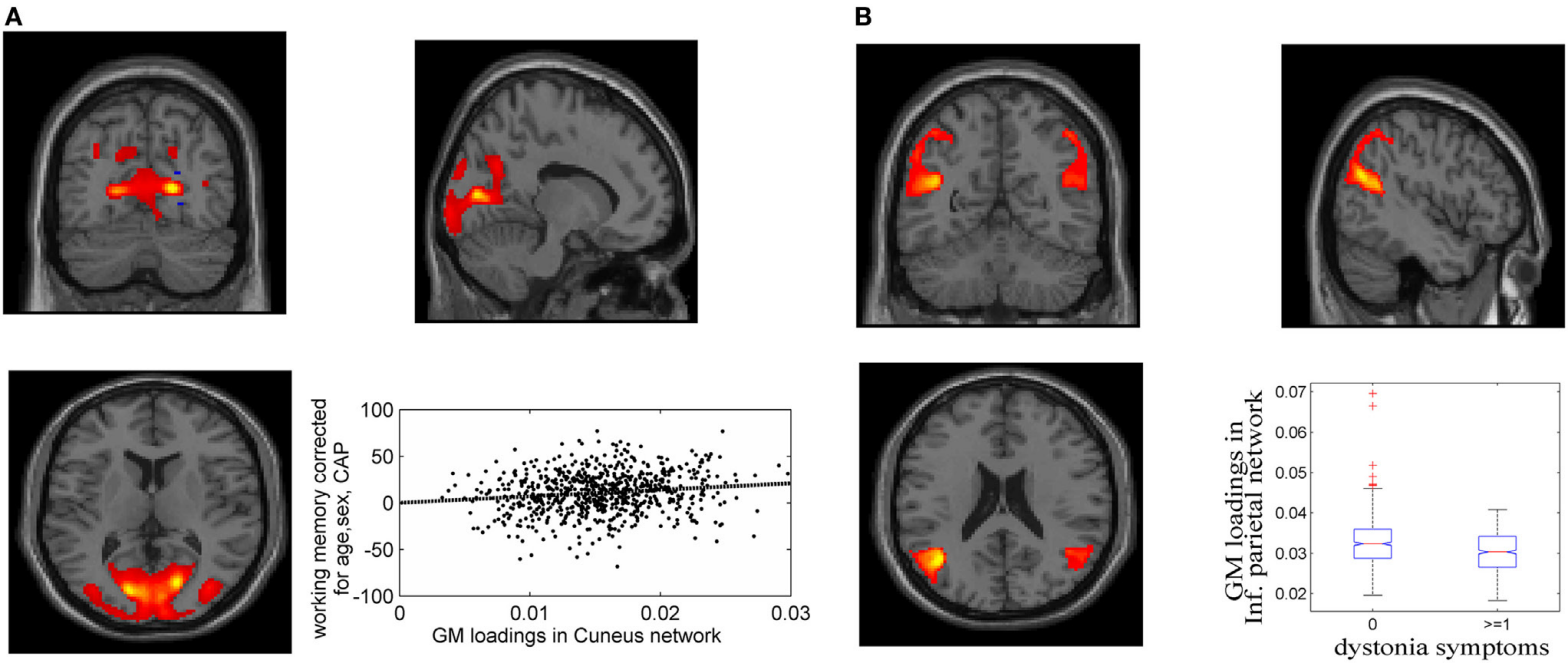
Gray matter (GM) components associated with working memory/attention and dystonia after controlling for disease burden. **(A)** GM component containing cuneus, lingual gyrus, and middle occipital gyrus, and its association with working memory/attention. **(B)** GM component highlighting bilateral inferior parietal and superior/middle temporal gyri, and its association with dystonia. The GM loadings are relative values without unit.

Fifteen GM components were extracted (see Supplementary Material), one of which was a typical artifact forming a ring around brain [as demonstrated by Chen et al. (37)]. This component was thus removed from further analyses. As expected, none of the GM components were related to CAP. The association tests with cognitive and motor functioning revealed a GM component (Figure 1A), mainly in cuneus, lingual gyrus, and middle occipital gyrus, that was significantly related to working memory and attention (*p* = 1.39 × 10^−4^ uncorrected, passing FDR correction). Higher GM volume in this component was related to better attention and working memory performance, explaining 1.7% of the variance after controlling for age, sex and CAP, as shown in Figure 1A (CAP explained 15.7%). Another GM component, mainly in bilateral inferior parietal and superior/middle temporal regions, was significantly related to dystonia (Figure 1B; logistic regression *p* = 2.34 × 10^−4^ uncorrected); prodromal participants with at least one dystonia sign had significantly lower GM volume in this network (Cohen’s *d* = 0.47, *p* = 2.37 × 10^−4^).

### GM and SNPs (Full Genomic Data and HD Pathway)

**Figure 2:**
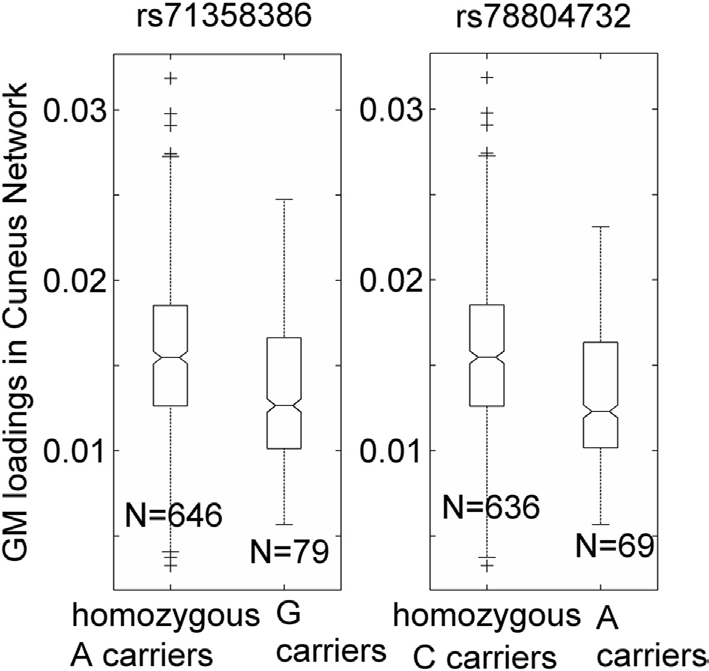
Association of two single-nucleotide polymorphisms, rs71358386 and rs78804732, with a cuneus gray matter (GM) component. GM loadings are relative values without unit. In the box plots, the middle line is the median value, the top and bottom of each box are the 25th and 75th percentile values, the whiskers extend from the ends of the interquartile to the further values within 1.5 times the interquartile, and plus (+) signs show values that are more than 1.5 times the interquartile range away from the top or bottom of the box. The plot of medium and 25/75th percentile presents a similar overall pattern as the mean and standard deviation in these data.

Over one million SNPs were tested for associations with GM variation in the aforementioned two components, and none exhibited a genomic significant association passing FDR correction. Similarly, no significant genomic associations with cognitive or motor functioning variables were observed. In our separate analysis of SNPs in 310 HD pathway genes, only one SNP (rs71358386 in *NCOR1)* was significantly associated with GM in the cuneus component (*p* = 2.38 × 10^−5^, passing FDR), with minor allele G being negatively linked to GM volume. For this SNP, 636 participants were homozygous major allele (A) carriers, 77 were heterozygous, and 2 were homozygous minor allele (G) carriers. We pooled the heterozygous and homozygous minor allele carriers together and computed the difference between minor allele carriers and homozygous major allele carriers. The difference was significant (*p* < 1.56 × 10^−5^ for the two-sample *t*-test and *p* < 6.42 × 10^−6^ for the Wilcoxon rank test), with a Cohen’s *d* of 0.53 (Figure 2). Interestingly, another SNP (rs78804732 in *ADORA2B*) was in strong LD with rs71358386 (*r* = 0.91). This SNP was also significantly associated with GM in the cuneus component (*p* = 1.51 × 10^−5^), with minor allele A being linked to lower GM volume and A carriers having significantly lower GM volume than major allele C carriers (Cohen’s *d* = 0.59; *p* < 8.0 × 10^−6^ for the two-samples *t*-test; *p* < 3.48 × 10^−6^ for the Wilcoxon rank test; Figure 2). These two SNPs were also nominally associated with GM in the inferior parietal component (*p* = 0.02 and *p* = 0.04, respectively), with minor alleles being linked to lower GM volume. An extended analysis on these two SNPs provided some promising but not strictly significant results, and we reported them in the Supplementary Material for the interest of readers. At the pathway level, no SNPs were significantly associated with motor or cognitive functioning, though these two SNPs were marginally associated with overall motor functioning (*p* = 0.05, not passing FDR correction), with more minor alleles being linked to greater motor dysfunction. To obtain an intuitive effect size, we assessed these SNPs’ effects on TMS and found that one minor allele copy was associated with an increase of 0.20 U in TMS score after controlling for age, sex, CAP, and MDS.

### Candidate SNP Analyses

**Figure 3:**
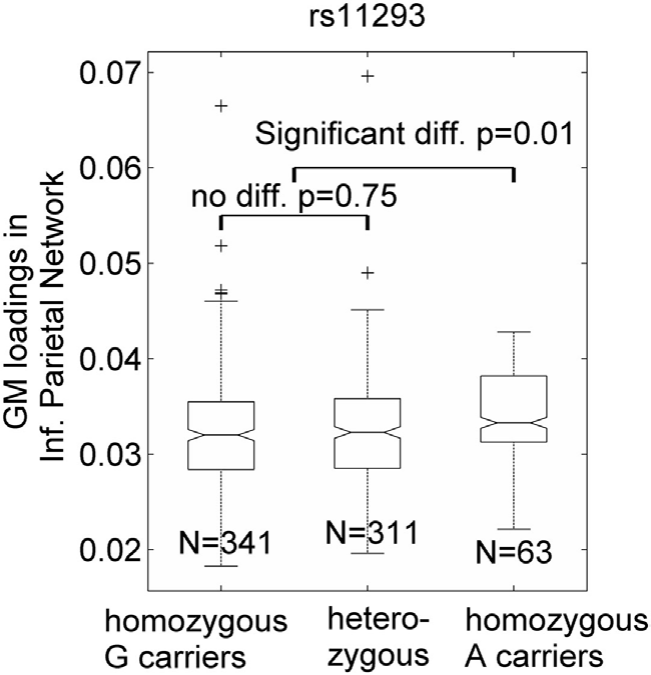
Single-nucleotide polymorphism rs11293’s association with an inferior parietal gray matter (GM) component. GM loadings are relative values without unit. In the box plots, the middle line is the median value, the top and bottom of each box are the 25th and 75th percentile values, the whiskers extend from the ends of the interquartile to the further values within 1.5 times the interquartile, and plus (+) signs show values that are more than 1.5 times the interquartile range away from the top or bottom of the box. The plot of medium and 25/75th percentile presents a similar overall pattern as the mean and standard deviation in these data.

Seven SNPs in LD with rs2140734 in chromosome 15 showed a marginal connection to GM in the inferior parietal network in the regression model (*p* = 0.06–0.09, not significant); greater minor allele number was linked to increased GM in the network. Further ANOVA tests revealed that the main driver of the association was the homozygous minor allele carrier group. As shown in Figure 3 using the example of rs11293, there was no difference between homozygous major allele G carriers and heterozygous carriers (*p* = 0.75), but homozygous minor allele A carriers had significantly higher GM than the other groups (*p* = 0.01, no multiple comparison correction was applied due to near identical patterns among the seven SNPs). This SNP was also negatively related to overall motor function (*p* = 0.01), indicating an association with better motor performance. To obtain an intuitive effect size, we assessed its effect on TMS, and found that one minor allele copy was associated with a TMS score decrease of 0.14 U after controlling for age, sex, CAP, and MDS. No connections with GM, cognition or motor functioning were observed for SNPs in LD with rs1037699 on chromosome 8.

## Discussion

Gray matter and motor and cognitive functioning show significant prodromal decline in HD (11, 15, 25, 38–41). Our results first confirmed that variation in these domains relates significantly to CAP, a metric reflecting disease burden and based on CAG mutation and exposure time (age) (13, 42). Individuals with more CAG repeats are likely to develop symptoms more rapidly and be diagnosed at younger ages. However, our results agree with previous work showing that a considerable amount of variance in prodromal functional decline is beyond this disease burden (3, 20). After regressing out CAP effects, two GM components yielded significant associations with working memory/attention and dystonia, respectively, though the variance accounted for was relatively small compared to CAP influence (about one tenth). Nonetheless, this is an exciting finding; the *HTT* CAG expansion is a causal mutation associated with HD, and age has widely known effects on GM variation and clinical functioning in both prodromal/HD patients and healthy individuals. Thus, modest residual effects are to be expected. As modifiers of disease progression, symptoms, and onset continue to be discovered, the potential for promising gene therapies increases as well. Such therapies could eventually target multiple modifiers with modest individual effects but substantial combined influence on progression. These findings reinforce evidence that the disease burden from CAG mutation and age does not explain all observed variance in prodromal disease progression and clinical onset (3, 20), and further suggest that GM variability may be a useful phenotype for examining genetic factors that account for unexplained variability in HD progression and onset.

Better performance on *working memory and attention* tasks was associated with higher GM volume in a component that included *cuneus, lingual gyrus*, and *middle occipital gyrus*. Structural changes in occipital regions have been consistently documented in prodromal and diagnosed HD, albeit overshadowed by caudate and putamen effects (8, 40, 43–46). Our findings are mirrored by a study staging cortical thinning across the prodrome, in which visual cortical regions were among the earliest and most severely affected regions, and cortical thinning in these regions was associated with lower scores on Stroop Color, Stroop Word, and SDMT (45). Similarly, a PREDICT-HD study investigating neuroanatomical correlates of five cognitive functions also reported that occipital cortical thickness was associated with letter-number sequencing working memory, as well as SDMT performance (8). In prodromal and diagnosed patients (relative to controls), TRACK-HD also reported reduced occipital cortical thickness, which was associated with poorer performance on the SDMT, Stroop Word test, and TMTA (44). Taken together, these findings highlight cuneus, lingual, and occipital abnormalities in prodromal and diagnosed patients, and indicate that these aberrations may influence cognitive performance. Our findings support these previous associations, and further suggest that they may be partially modulated by factors outside of *HTT* CAG repeat number and age.

*Dystonia* is a common symptom of HD manifesting at varying degrees of severity (47). In our cohort, dystonia signs were associated with reduced GM in a component containing *inferior parietal* and *middle and superior temporal* regions, after controlling for CAP. Inferior parietal areas interface with other sensorimotor regions to promote motor planning and initiation (48), and show increased activation before self-initiated movements (49). Inferior parietal GM loss has been reported in prodromal patients and is consistently observed in diagnosed HD (45, 46, 50), and has been further linked to abnormal eye movement (50). A meta-analysis of HD voxel-based morphometry studies identified brain clusters associated with motor symptoms, grouping inferior parietal together with precentral gyrus, primary motor, postcentral gyrus, and somatosensory cortex; these regions were more strongly related to motor functioning than the caudate (46). As for superior temporal gyrus, a smaller prodromal study (*N* = 325) associated bilateral superior temporal cortex with motor timing precision, and found that it was the greatest structural contributor to performance outside of the striatum and middle frontal cortex (8). These studies emphasize the importance of temporal and parietal regions in movement-related tasks in both healthy controls and prodromal and diagnosed HD patients. Our results reinforce these findings, and the removal of CAP effects in our analyses further suggests that a portion of these effects relates to factors outside of the disease-determining *HTT* mutation.

Frontal and striatal abnormalities are the most robust and commonly reported effects in HD, and these regions are heavily involved in cognitive and motor functioning. Our findings reflect brain structural influences on cognition and movement that are not accounted for by disease burden. It is thus unsurprising that the striatum and frontal lobe were not key contributors to the effects we report. Alternatively, our results pinpoint occipital, parietal, and temporal regions of the brain that comprise networks important for attention, working memory, and planned movement. These areas often work in concert with the frontal lobe and striatum to promote cognitive and motor functioning. In this large prodromal cohort, these regions appear to contribute to prodromal clinical functioning in a manner that is independent of *HTT* CAG influence.

The genome-wide association test did not produce significant results, which is not particularly surprising since HD is a rare disorder and genomic tests require very large sample sizes to balance multiple comparison corrections and small effect sizes. Similar to studies of genetic modifiers of motor onset time (5, 20), some true genetic effects may be missed due to strict genome-wide significance thresholds. The HD pathway-based genetic association analysis leveraged prior knowledge of gene functions and their involvement in HD. Therefore, these findings fit into the double-hit phenomena in which gene functions are known to contribute to disease pathogenesis, and changes in these genes are also related to GM variation that contributes to prodromal symptoms and cognitive decline. Thus, these genetic variants have an increased likelihood of affecting disease progression.

We observed two SNPs in strong LD but located in two different genes (*NCOR1* and *ADORA2B*, 54k base pairs apart) that were associated with GM variations. In fact, SNP rs71358386 in *NCOR1* regulates expression of *ADORA2B* in various tissues based on the GTEx database^3^ (51). In our cohort, minor allele carriers of the two SNPs showed significant occipital GM reduction and some level of reduction in inferior parietal regions, as well as marginally higher motor dysfunction. *NCOR1* is part of the HD pathway and encodes the protein nuclear receptor corepressor 1, which mediates transcriptional repression of thyroid-hormone and retinoic acid receptors. This protein reportedly interacts with mutant HTT (52, 53) to alter nuclear receptor function and is also differentially located in patient brain tissue (53, 54). *ADORA2B* encodes adenosine receptor subtype A2B, a protein that interacts with netrin-1, which is involved in axon elongation. Currently, *ADORA2B* is not part of the HD pathway, although *ADORA2A* is (55–57). *ADORA2A* and *ADORA2B* are two of four human genes that encode adenosine receptors that increase cyclic adenosine monophosphate (58), which is important for signal transduction and other biochemical processes (59). We cannot currently establish these SNPs as true causal mutations, and further investigation of the molecular, cellular, and functional impact of these genes is warranted.

In addition, within two candidate regions selected based on their significant effects on clinical onset time (20), our results revealed that SNPs in ch15q13.3 (*MTMR10* and *FAN1* genes) affected GM in prodromal participants; homozygous minor allele carriers had higher GM densities in the inferior parietal component. Given the negative link between GM volume in this component and dystonia symptoms and overall motor functioning, this minor allele has a protective effect on prodromal dystonia and motor dysfunction, with one minor allele copy being associated with a TMS score decrease of 0.14. Excitingly, this finding is in total agreement with the reported clinical onset delay attributed to these SNPs [the minor allele was associated with a 1.4-year onset delay (20)]. The possible mechanisms through which these genes influence disease progression have been elaborated upon by the GeM-HD study. Our results suggest that genetic variations outside of *HTT* are already altering GM in the prodromal phase, before the emergence of diagnosis-associated motor symptoms.

This investigation of extra-*HTT* genetic modifiers before clinical diagnosis represents a new direction for the development of treatments to prevent or delay this devastating disorder. Leveraging brain structural variation, which is likely more precise and subtle than clinical outcome changes, enhances power for identifying genetic modifiers. The findings of this study demonstrate that: (1) GM variation beyond CAG influence is associated with disease progression and manifests as early as the prodrome; (2) genetic modifiers of biologically measured GM volume are already exerting their effects during the prodromal phase; and (3) the accumulation of these effects across disease progression ultimately alters clinical onset time. Replication using an independent sample and follow-up studies manipulating cell lines or animal strains should be carried out to fully illuminate the mechanisms of these genetic modifiers. As a proof of concept, our findings suggest that studying brain structural variation beyond disease burden can be a very promising method for identifying genetic modifiers of HD progression. The limitations of this study include the following: (1) inclusion of some gene positive participants who may never be diagnosed with HD and thus may be healthy participants; (2) only linear relationships between GM, cognition, motor functioning, and genetic variations were tested; and (3) a longitudinal study on changes in GM, cognition, and motor functioning, as well as a carefully designed comparison with healthy controls, would help confirm the genetic effects reported here.

## Ethics Statement

All participants provided written, informed consent and were treated in accordance with protocols approved by each participating institution’s internal review board.

## Author Contributions

JL, JT, and VC have designed this study; JL, JC, and DL have conducted data analyses; JL and JC were involved in writing the first draft, and all authors contributed to reviewing and editing the final manuscript.

## Conflict of Interest Statement

The authors declare that the research was conducted in the absence of any commercial or financial relationships that could be construed as a potential conflict of interest.

## Footnotes

^1^https://analysistools.nci.nih.gov/LDlink/ (Accessed: April, 2017).

^2^http://www.fil.ion.ucl.ac.uk/spm/software/spm8/.

^3^https://gtexportal.org (Accessed: April, 2017).
