# Supplementary material for: Genetics Modulate Gray Matter Variation Beyond Disease Burden in Prodromal Huntington’s Disease
Source: Front Neurol. 2018 Mar 29;9:190. doi: 10.3389/fneur.2018.00190 (PMC5884935; doi:10.3389/fneur.2018.00190)
Supplement: Supplementary file 1 [file data_sheet_1.docx]

Supplementary information

A: Cognitive and motor variables:

SDMT = Symbol Digit Modalities Test. The SDMT measures working memory, complex scanning, and processing speed, and is an adaptation of the Wechsler Digit Symbol subtest [[1](#_ENREF_1), [2](#_ENREF_2)]. For SDMT, participants are provided with a key of symbols paired with numbers (at the top of the test page). On the same test sheet, a series of numbers are presented in a horizonal row, and the task is to fill in the symbols matching each number in the sequence as quickly and accurately as possible. Raw scores represent the number of correctly completed items within 90 seconds [[3](#_ENREF_3)].

Stroop Color = Stroop Color and Word Test – color condition. The color condition is a color identification task that measures basic attention, and is the first of three 45-second trials in the Stroop Color and Word Test [[4](#_ENREF_4)]. The task is to identify colors presented on stimulus cards, and raw scores reflect the number of correct responses [[5](#_ENREF_5)].

Stroop Word = Stroop Color and Word Test – word condition. For the Stroop Color and Word test word condition, participants are tasked with reading color names presented in black ink. The word condition is the second of three 45-second trials in the Stroop Color and Word Test [[4](#_ENREF_4)]. Like the color condition, the word condition measures basic attention, and raw scores represent the number of correct responses [[5](#_ENREF_5)].

Stroop Interference = Stroop Color and Word Test – interference condition. The Stroop Interference task is the third 45-second trial of the three-part Stroop Color and Word Test [[4](#_ENREF_4)]. For the Interference task, participants inhibit an overlearned response by naming the color that color-names are presented in rather than reading the color name itself (e.g., for the word “blue” printed in green ink, the correct response is “green”). Like the other two conditions, raw scores reflect the number of correct trial responses [[5](#_ENREF_5)].

TMTA = Trail Making Test, Part A. For TMTA, the task is to sequentially connect a series of numbered circles (e.g., 1-2-3-4) as quickly as possible. Raw scores reflect time (in seconds) taken to complete the task. Thus, higher scores reflect poorer performance [[6](#_ENREF_6), [7](#_ENREF_7)].

TMTB = Trail Making Test, Part B. For TMTB, participants are asked to connect consecutive numbers and letters in ascending/alphabetical order, alternating between numbers and letters (e.g., 1-A-2-B-3-C). Like TMTA, raw scores reflect completion time in seconds, and higher scores reflect poorer performance [[6](#_ENREF_6), [7](#_ENREF_7)].

TMS = total motor score (TMS) from the Unified Huntington Disease Rating Scale (UHDRS). TMS is a standardized measure of oculomotor function, dysarthria, chorea, dystonia, gait and postural stability, and is part of the UHDRS [[8](#_ENREF_8)].

Oculomotor = ocular subscale from the UHDRS. The ocular-motor subscale of the UHDRS assesses eye motion and tracking ability [[8](#_ENREF_8), [9](#_ENREF_9)].

Bradykinesia = bradykinesia subscale from the UHDRS. The bradykinesia subscale of the UHDRS measures the level of movement rigidity or slowness [[8](#_ENREF_8), [9](#_ENREF_9)].

Chorea = chorea subscale from the UHDRS. The chorea subscale of the UHDRS assesses the frequency and severity of abnormal involuntary movements in the face, mouth, trunk, and extremities. Chorea symptoms are scored from 0 (absent) to 4 (marked/prolonged) in each region, and the sum of the scores is the chorea-motor score [[8](#_ENREF_8), [9](#_ENREF_9)].

Dystonia = dystonia subscale from the UHDRS. The dystonia subscale from the UHDRS measures the occurrence and severity of involuntary muscle contractions and twisting in the trunk and extremities of the body. Like chorea, dystonia symptom scores range from 0-4 for each bodily region, and the sum of these scores is the dystonia score [[8](#_ENREF_8), [9](#_ENREF_9)].

B: Association test results for individual motor and cognitive variables

1) Eleven motor and cognitive variables were tested for association with CAP using a regression model: a cognitive or motor variable = age + sex + CAP. Poisson regression was used for motor variables and linear regression was used for cognitive variables, due to the distribution patterns of each variable. Table S1 lists the association p values and percentage of variance explained by the model. For individual variables, the association with CAP was significant (p < 0.01 for dystonia and p < 1e-11 for the others). These results are consistent with those reported in the main text using representative variables.

Table S2: individual variable associations with age, sex and CAP.

|  | Age_p | Sex_p | CAP_p | variance explained % |
| --- | --- | --- | --- | --- |
| TMS | 1.72E-02 | 2.40E-01 | <1E-15 | 18.36% |
| oculomotor | 1.37E-02 | 4.79E-02 | 5.95E-12 | 10.21% |
| bradykinesia | 7.25E-02 | 2.70E-02 | <1E-15 | 14.89% |
| chorea | 9.46E-02 | 1.74E-01 | <1E-15 | 9.32% |
| dystonia | 1.05E-01 | 6.48E-01 | 6.19E-03 | 2.22% |
| SDMT | 3.84E-02 | 4.54E-01 | <1E-15 | 21.45% |
| stroop_color | 1.86E-01 | 1.95E-01 | <1E-15 | 13.60% |
| stroop_word | 6.45E-01 | 5.95E-01 | <1E-15 | 11.96% |
| stroop_interference | <1E-15 | 4.49E-01 | <1E-15 | 15.24% |
| TMTA | 3.51E-02 | 9.25E-01 | <1E-15 | 13.29% |
| TMTB | 2.20E-01 | 7.38E-01 | <1E-15 | 14.88% |

2) Association of individual motor and cognitive variables with GM components was tested using a regression model: a cognitive or motor variable = age + sex + CAP + GM network loadings. Poisson regression was used for motor variables and linear regression was used for cognitive variables. One GM component out of 15 represented artifacts and was not further analyzed. Table S2 lists the association p values (uncorrected) and bold indicates p values passing FDR correction. GM component 6 consists of cuneus, lingual gyrus, and middle occipital gyrus, and is associated with overall working memory/attention performance (see main text). Here, this component was associated with SDMT, Stroop Color, and Stroop Interference. Component 11 includes GM in bilateral inferior parietal and superior/middle temporal gyri, and was associated with the binary dystonia variable in the main text. Here, it was associated with original dystonia scores. GM component 14 consists of bilateral insula and superior temporal gyrus. This component was associated with SDMT, but not with any other individual cognitive measure nor any representative measure from the main text. A further association test between component 14 GM loadings and genomic SNPs, pathway SNPs and candidate SNPs also revealed no significant SNP association. Due to the relatively sparse association, we believe further validation of the component 14 result is necessary. Overall, the results from individual variables are highly consistent with those derived from representative variables reported in the main text.

Table S3: Association p values of individual variables with GM components

| GM network | TMS | oculo | brady | chorea | dystonia | SDMT | Stroop  color | Stroop  word | Stroop  Inter-ference | TMTA | TMTB |
| --- | --- | --- | --- | --- | --- | --- | --- | --- | --- | --- | --- |
| 1 | 7.96E-01 | 3.67E-01 | 8.31E-01 | 6.03E-01 | 4.38E-01 | 7.60E-01 | 6.59E-01 | 6.11E-01 | 4.22E-01 | 3.93E-01 | 5.47E-01 |
| 2 | 9.99E-01 | 5.13E-01 | 2.26E-01 | 4.27E-01 | 2.34E-01 | 4.38E-01 | 2.74E-01 | 8.78E-01 | 8.55E-01 | 3.01E-01 | 4.15E-02 |
| 3 | 9.91E-01 | 3.10E-01 | 9.30E-01 | 1.99E-01 | 7.37E-01 | 5.09E-01 | 7.79E-01 | 7.99E-01 | 4.39E-01 | 4.13E-01 | 7.24E-01 |
| 4 | 8.70E-01 | 6.87E-01 | 3.75E-01 | 7.47E-01 | 3.94E-01 | 2.56E-01 | 2.93E-01 | 2.09E-01 | 3.15E-02 | 8.27E-01 | 1.21E-02 |
| 5 | 9.63E-01 | 8.41E-01 | 6.59E-01 | 9.30E-01 | 3.67E-01 | 9.52E-01 | 8.63E-01 | 5.87E-01 | 5.94E-01 | 7.00E-01 | 2.54E-01 |
| 6* | 5.48E-03 | 9.79E-02 | 3.94E-02 | 5.02E-03 | 3.40E-01 | **1.31E-04** | **2.34E-04** | 9.07E-03 | **1.16E-04** | 6.73E-03 | 3.64E-03 |
| 7 | 9.12E-01 | 8.59E-01 | 7.38E-01 | 4.33E-01 | 8.34E-01 | 2.86E-01 | 4.02E-01 | 6.43E-01 | 6.98E-02 | 5.09E-01 | 3.29E-01 |
| 8 | 5.99E-01 | 2.39E-01 | 8.92E-01 | 8.06E-01 | 8.41E-01 | 4.96E-01 | 1.55E-01 | 4.18E-01 | 3.97E-02 | 7.65E-01 | 3.85E-02 |
| 10 | 8.71E-02 | 5.77E-01 | 2.13E-02 | 4.83E-01 | 7.27E-01 | 5.75E-02 | 1.81E-01 | 2.55E-02 | 2.73E-01 | 5.41E-01 | 1.41E-01 |
| 11** | 1.22E-02 | 2.49E-01 | 9.07E-02 | 2.16E-02 | **2.54E-06** | 2.44E-01 | 3.55E-01 | 6.93E-01 | 6.72E-01 | 6.21E-01 | 3.26E-01 |
| 12 | 5.01E-01 | 5.39E-01 | 2.32E-01 | 5.61E-01 | 1.54E-02 | 1.05E-01 | 8.92E-01 | 3.31E-01 | 6.52E-01 | 6.99E-02 | 1.36E-01 |
| 13 | 4.04E-01 | 6.96E-01 | 2.10E-01 | 6.00E-01 | 5.30E-01 | 6.46E-01 | 5.15E-01 | 6.21E-01 | 2.58E-01 | 6.26E-01 | 2.71E-01 |
| 14*** | 1.73E-02 | 1.02E-01 | 2.86E-02 | 2.09E-01 | 1.63E-02 | **2.19E-04** | 5.69E-01 | 2.24E-01 | 2.65E-01 | 1.71E-02 | 1.06E-02 |
| 15 | 6.70E-01 | 8.46E-01 | 6.91E-01 | 6.86E-01 | 4.70E-01 | 6.10E-01 | 5.93E-01 | 6.26E-01 | 2.46E-01 | 2.48E-01 | 9.77E-01 |

*cuneus, lingual gyrus, middle occipital gyrus component. **bilateral inferior parietal and superior/middle temporal network. ***bilateral insula and superior temporal gyrus component.

3) Association of motor and cognitive variables with SNPs was tested using a regression model: a motor or cognition variable = age + sex + CAP + SNP + top ten MDS scores. Poisson regression was used for motor variables and linear regression was used for cognitive variables. The tests, genomic level and pathway level, were FDR corrected at p < 0.05 for all tested variables and SNPs. No significant associations were observed. For candidate SNP analyses, SNPs in chromosome 15 (in high LD) showed associations with motor variables. Using rs11293 as an example, this SNP was related to TMS (p = 0.02), oculomotor (p = 0.02), and chorea (p = 0.05), with more minor alleles being linked to lower scores; this is consistent with the overall motor function association reported in the main text. Three identical SNPs in chromosome 8, (rs16869295, rs11777942, and rs11778107) were associated with chorea score (p = 0.003) but not with other scores. Here, greater minor allele numbers were associated with higher chorea scores, in keeping with a hastening of onset that is in line with previous reports [[10](#_ENREF_10)].

4) In an extended analysis focusing on the SNP rs71358386 in *NCOR1* (similar results were obtained for rs78804732 in *ADORA2B*), we found it was marginally correlated with one GM component consisting of caudate, insula, and superior temporal gyrus (STG) (p = 0.069, r = -0.065) and positively related to overall motor function (p = 0.05) and dystonia (p = 0.04). Very encouragingly, the caudate/insula/STG component showed a negative relationship with overall motor function (p = 0.02) and problem-solving processing (p = 0.007). Even though these p values did not pass FDR correction, the overall association pattern is very appealing; i.e., greater minor allele numbers are linked to less GM volume in the caudate/insula/STP component as well as higher motor scores and dystonia signs, and less GM in this component is linked to higher motor scores and slower problem-solving processing. This result warrants further exploration.

C: Supplementary figures


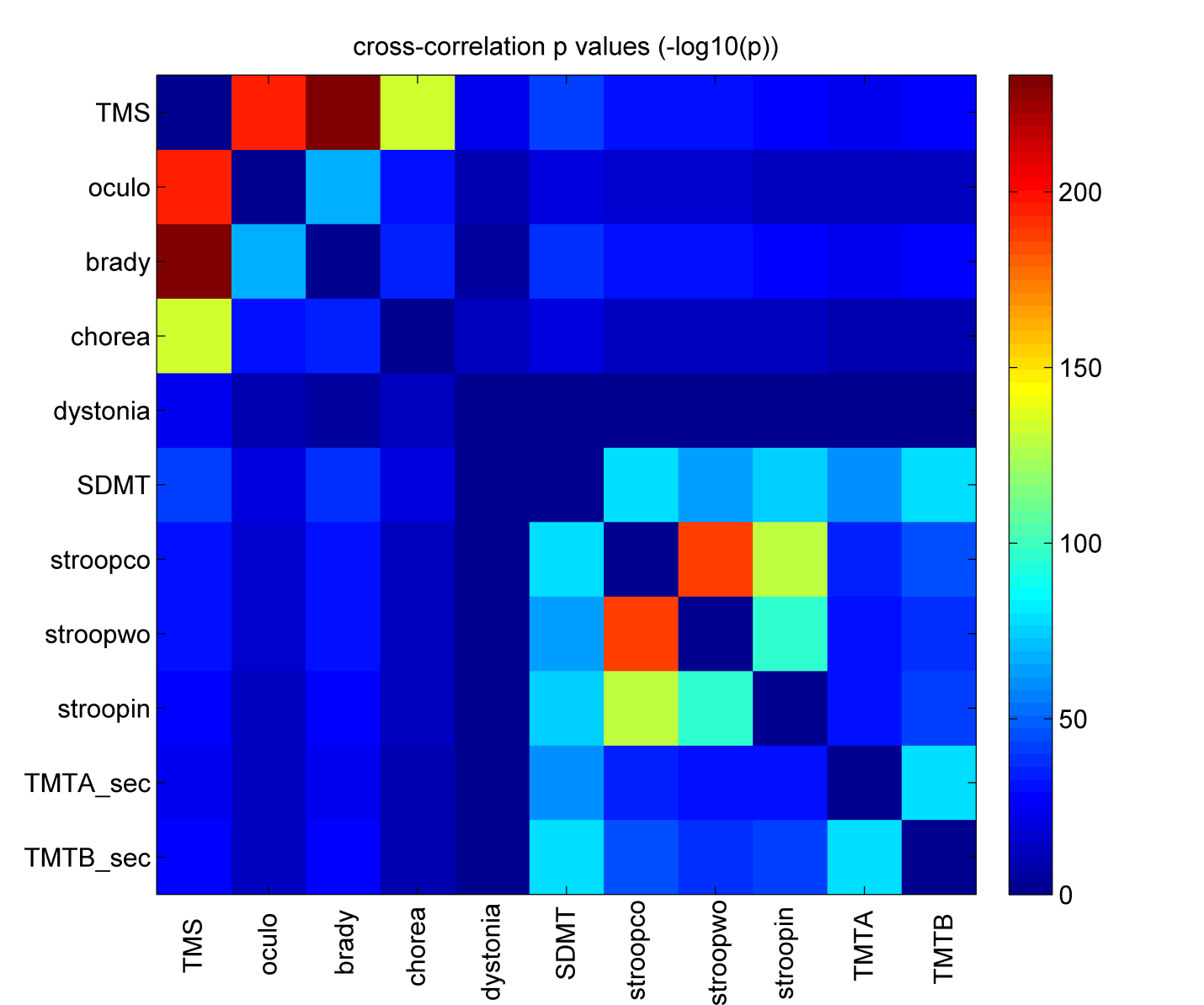


Figure S1. Cross correlation among cognitive and motor function variables. The diagonal auto-correlation was ignored.


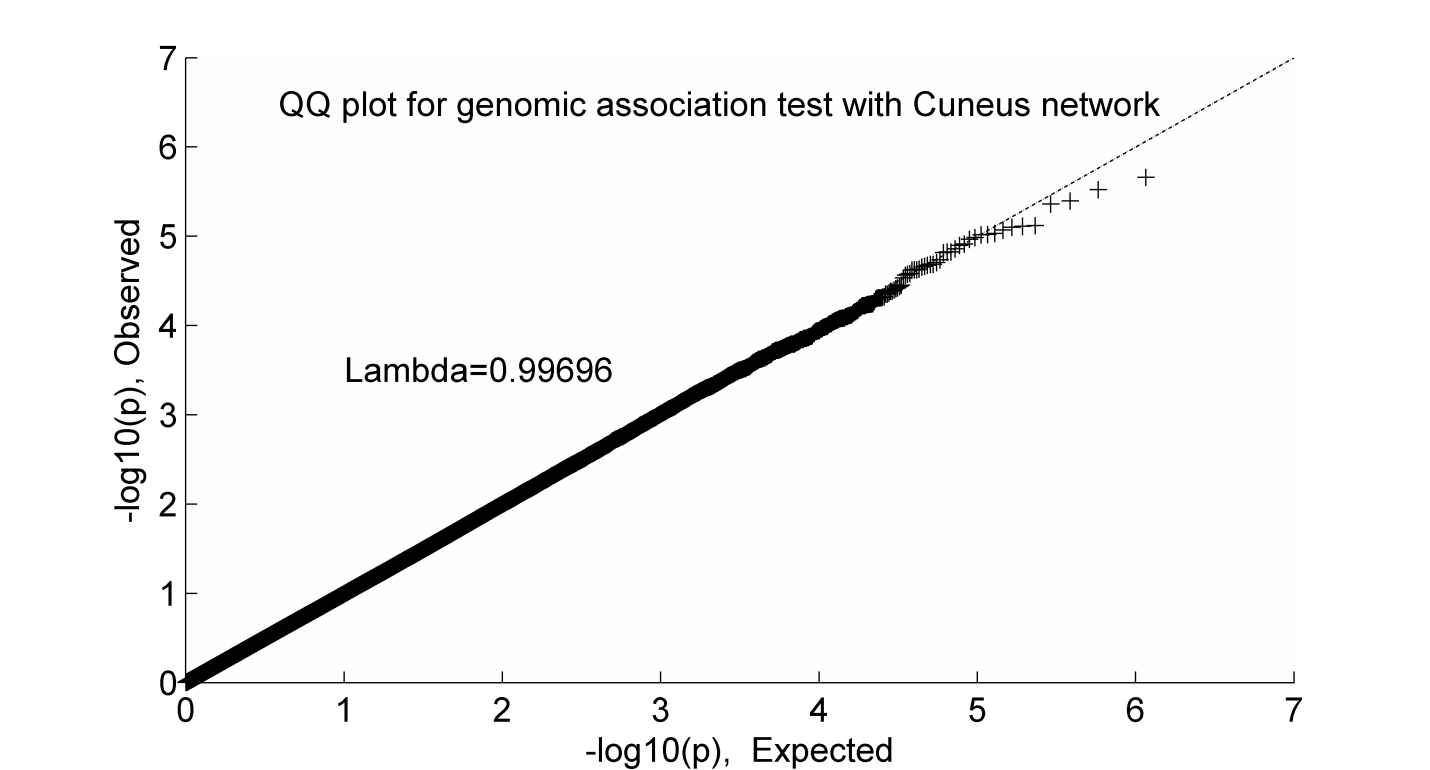
 A)
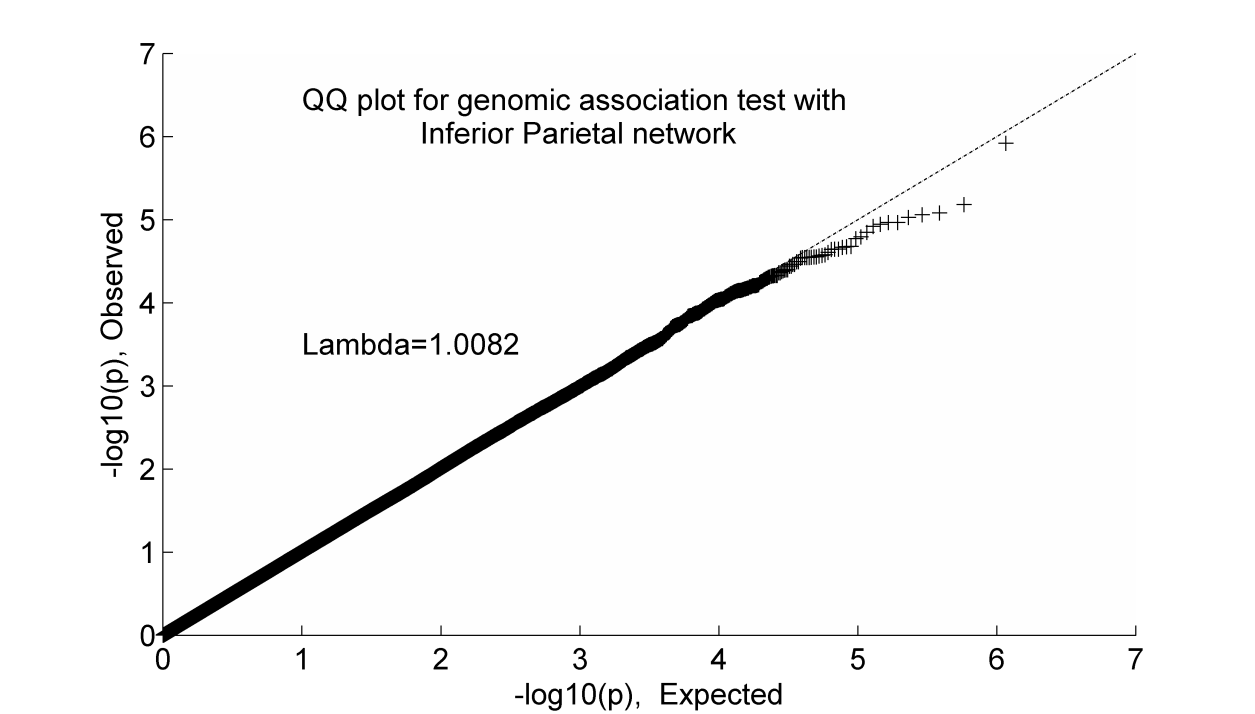
 B)
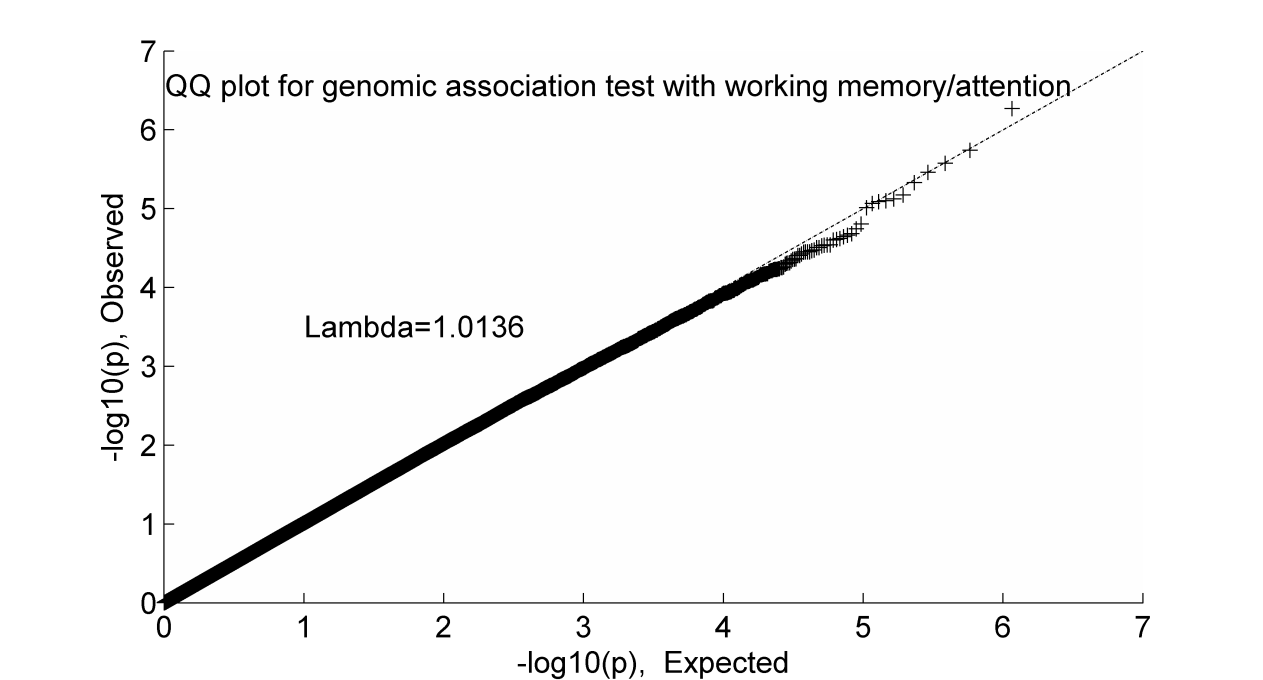
 C)
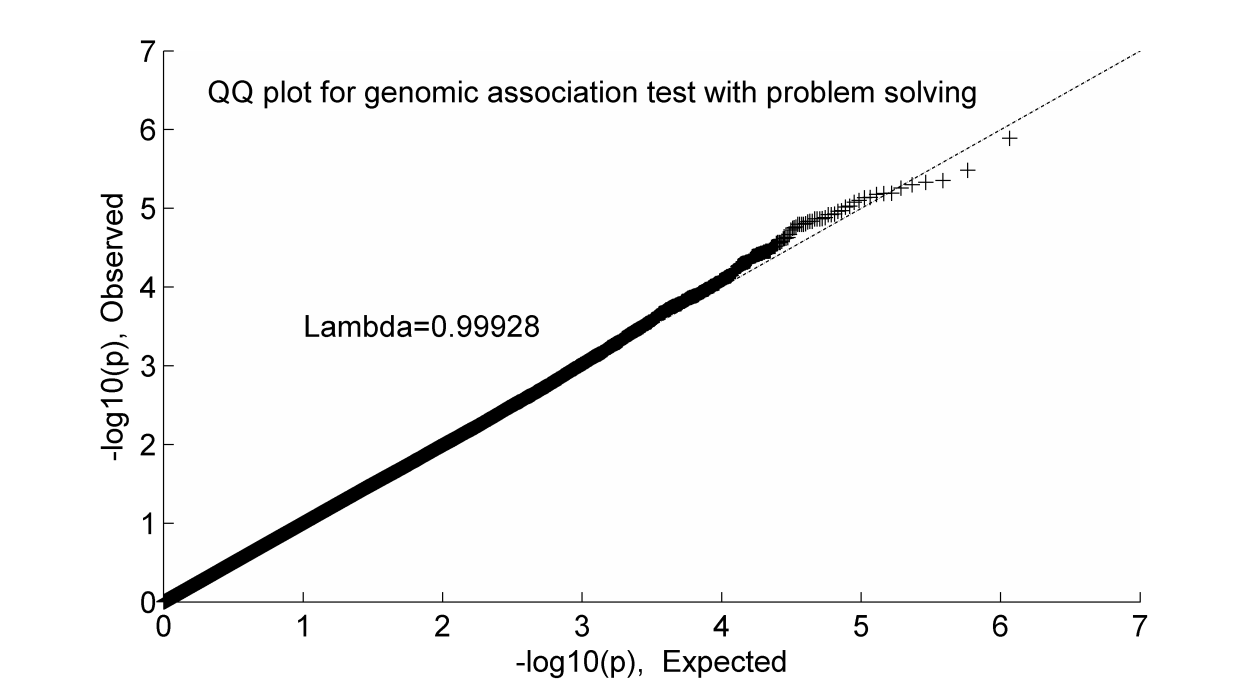
 D)
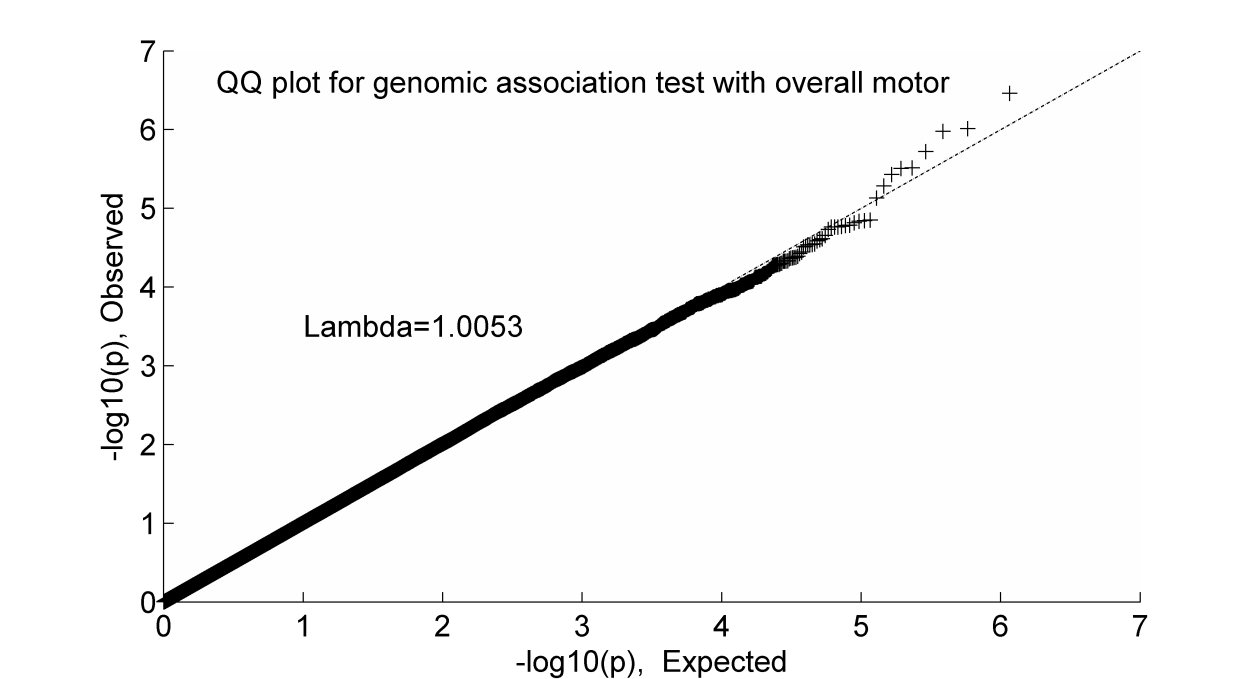
 E)
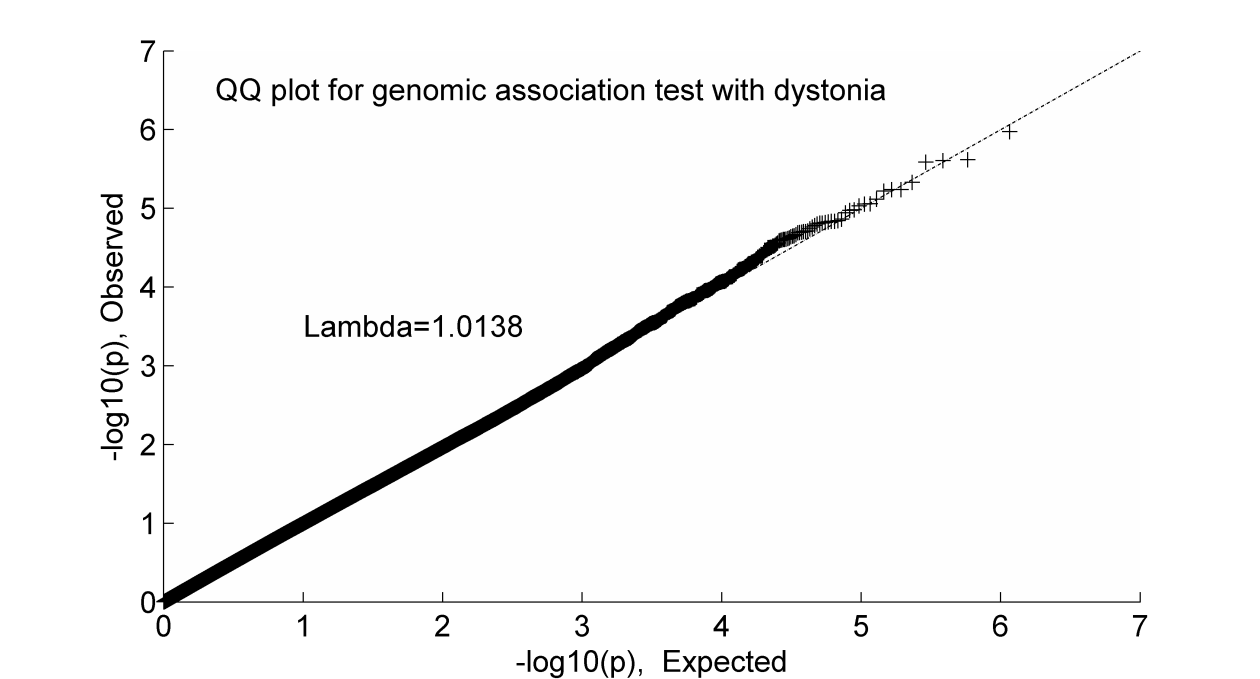
 F)

Figure S2. QQ plots for genomic association tests with A: cuneus GM component, B: inferior parietal GM component, C: working memory/attention, D: problem solving, E: overall motor function, and F: dystonia.

Figures of all GM components (|Z|>2.5)


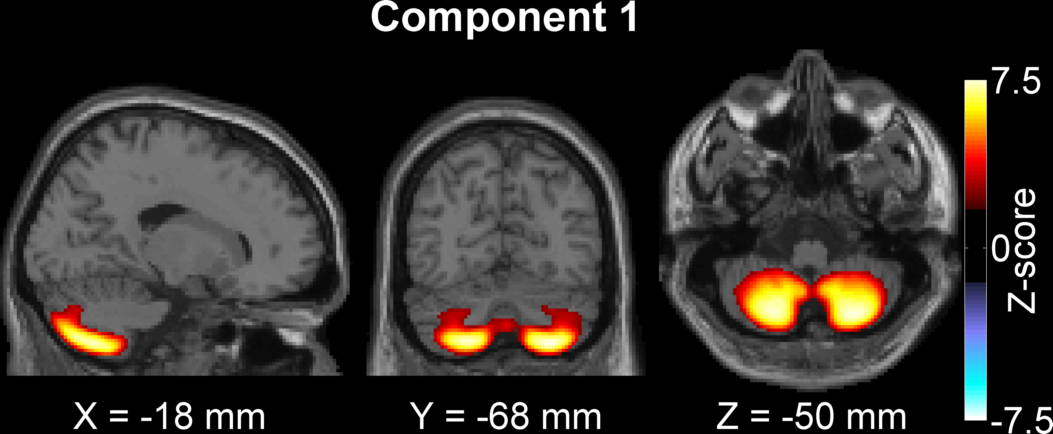

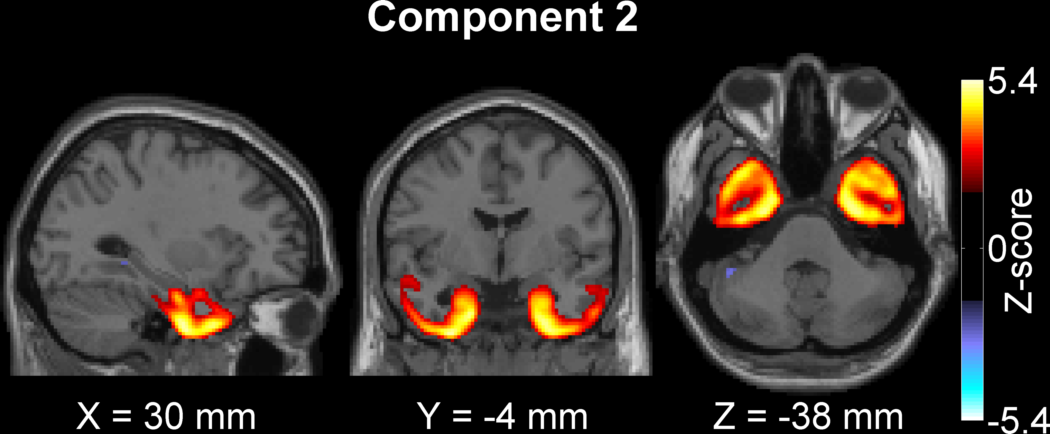

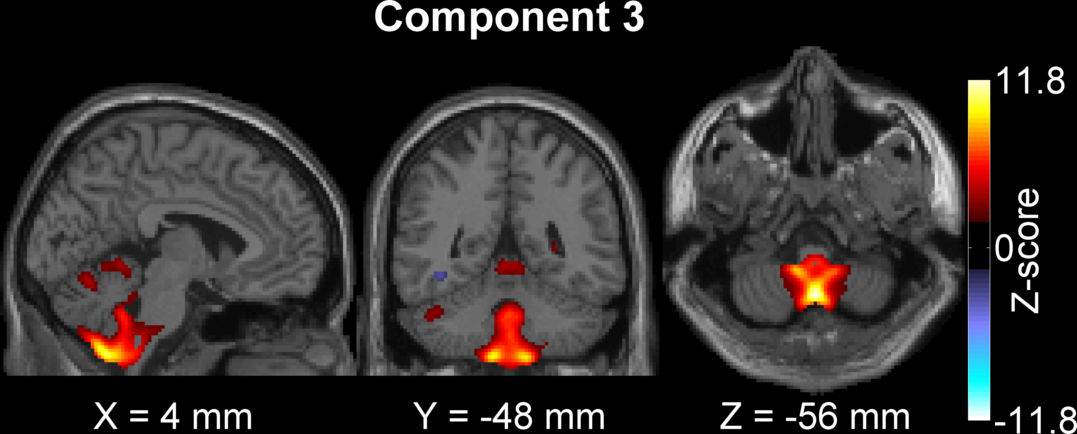


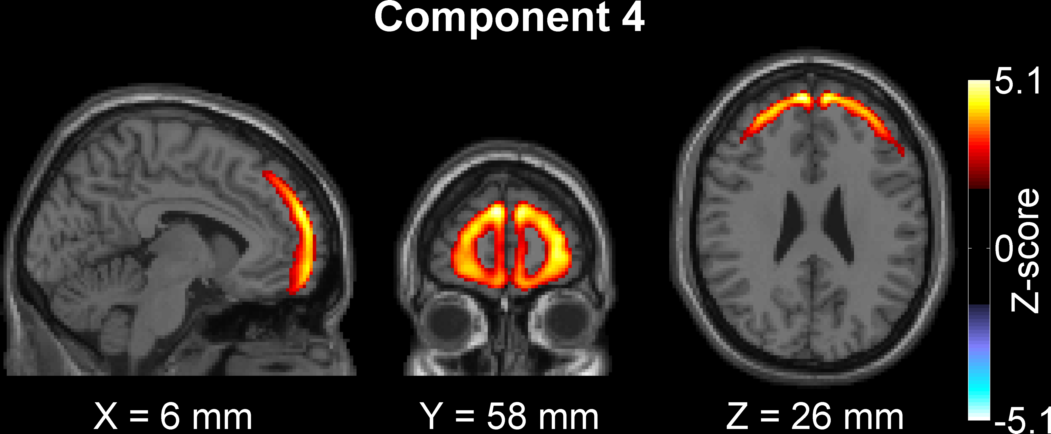

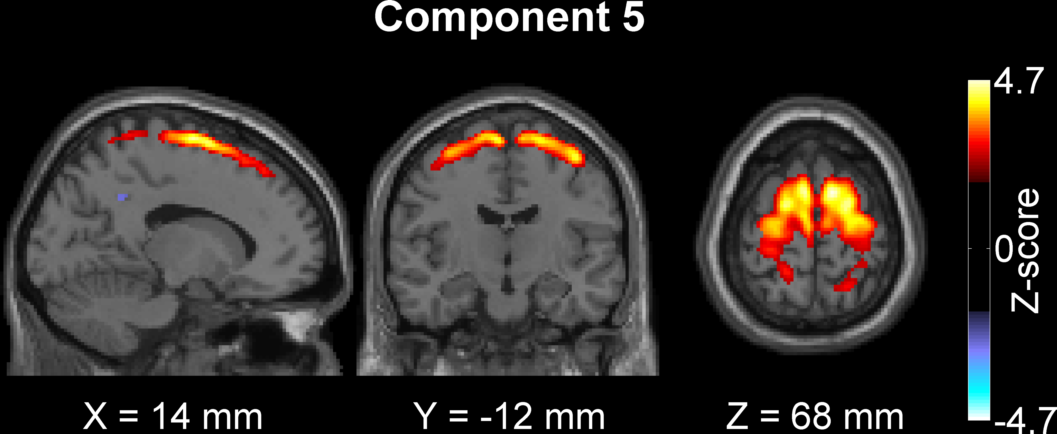

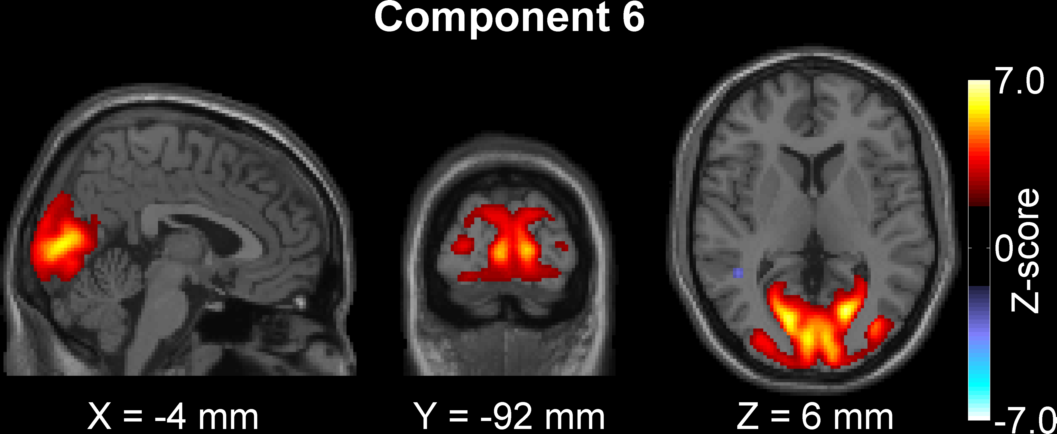


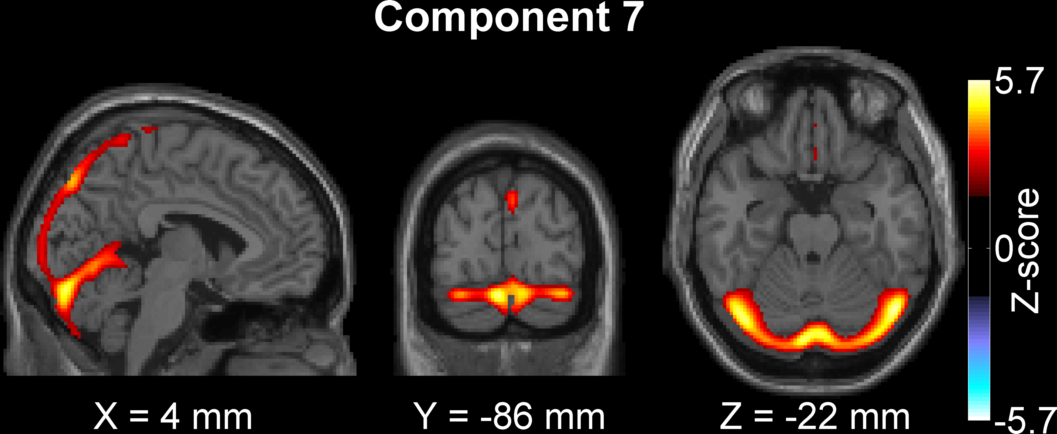

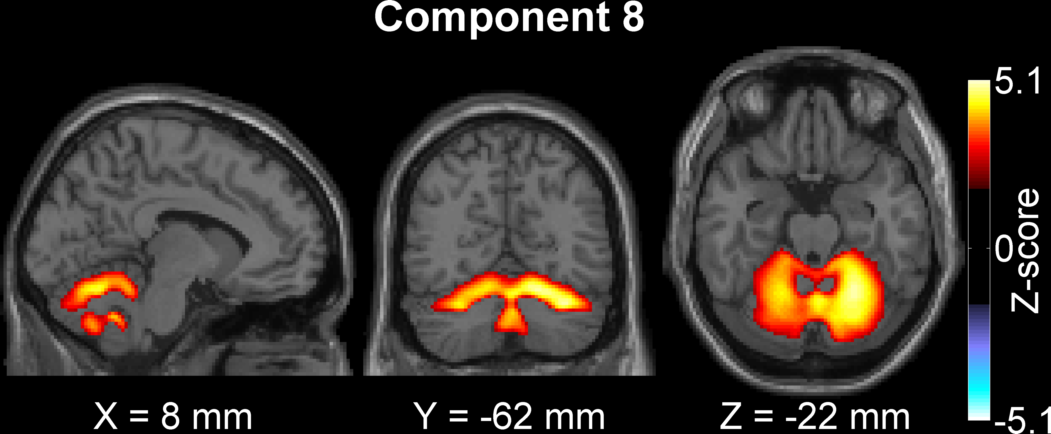

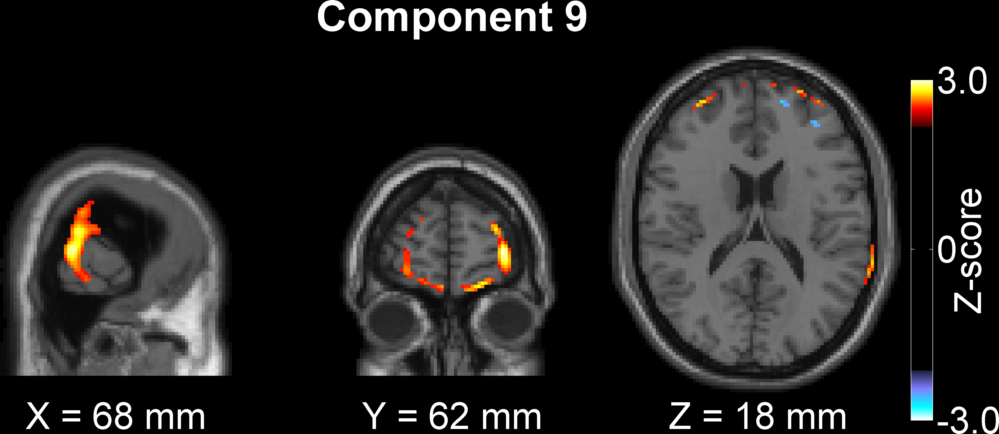


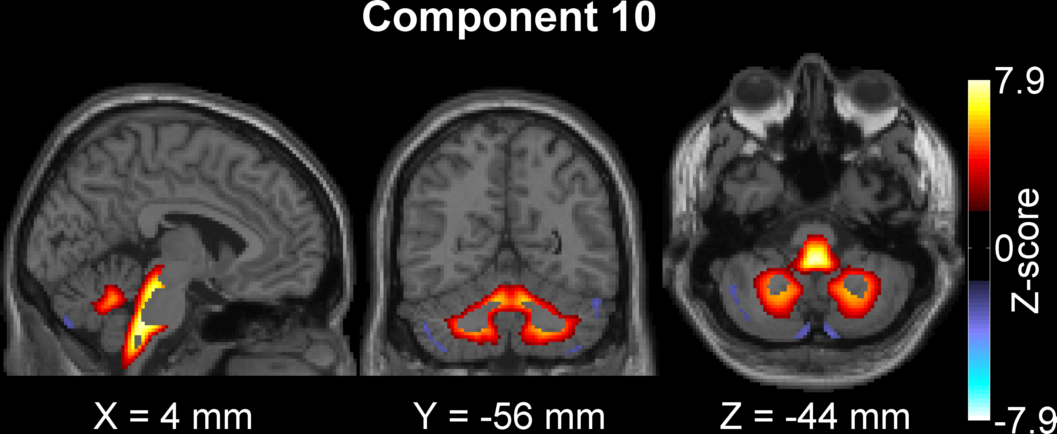

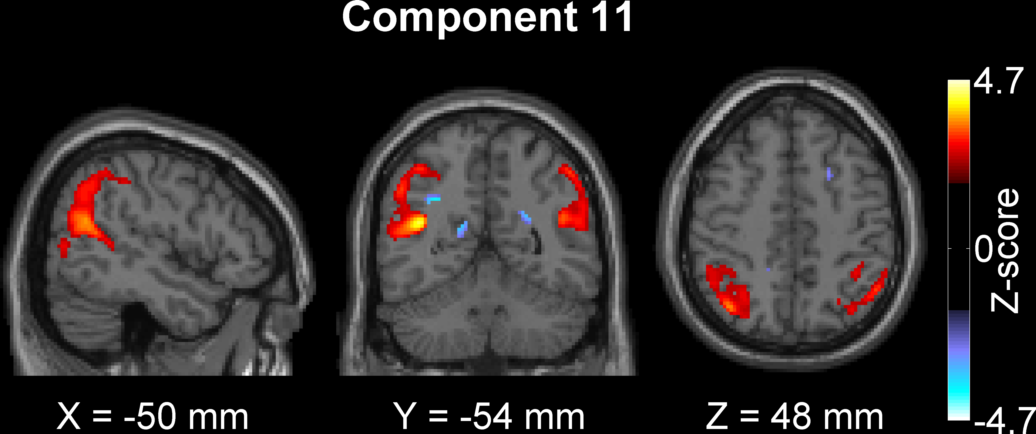

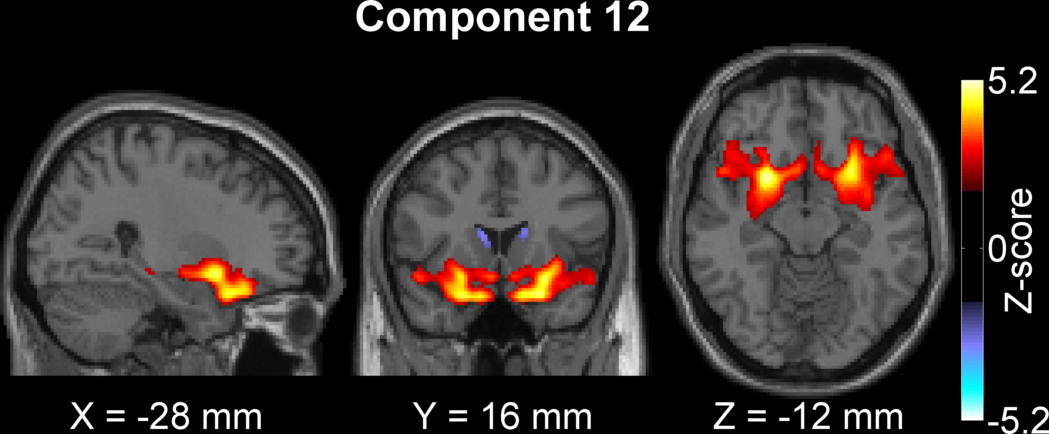


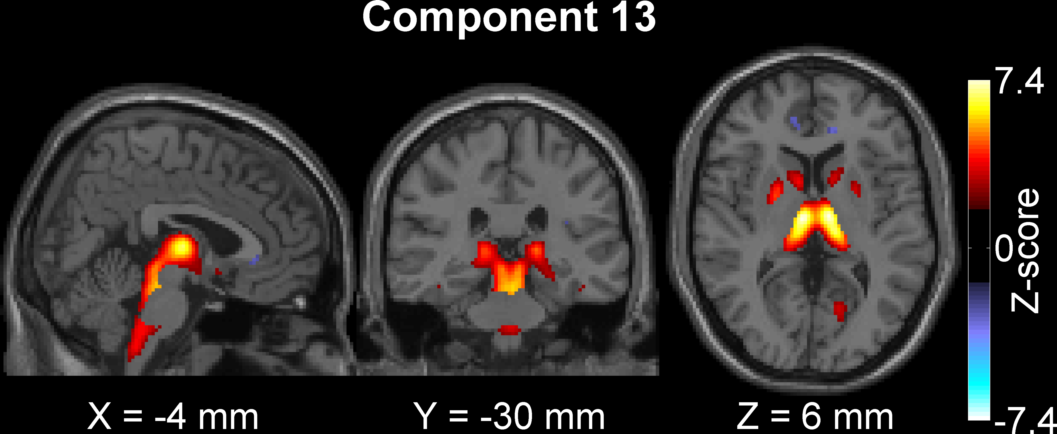

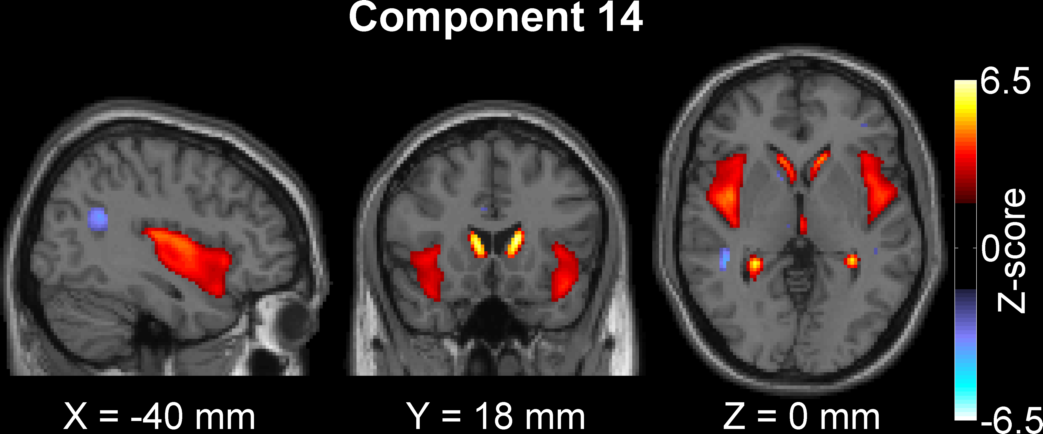

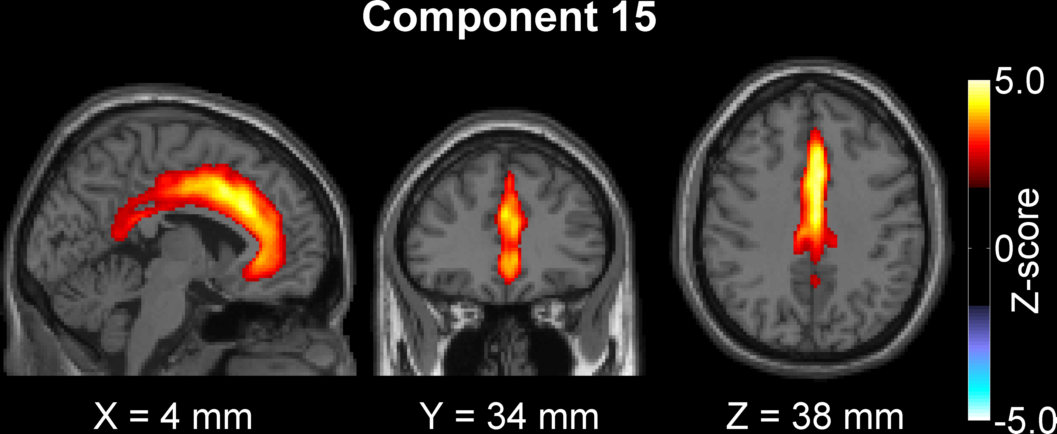


References

1. Wechsler, D., *Manual for the Wechsler Adult Intelligence Scale - Revised*, 1981, Psychological Corporation. World Health Organization (1988): New York, NY.

2. Lezak, M.D., D. Howieson, and D. Loring, *Neuropsychological assessment*, 2004, Oxford University Press: New York.

3. Smith, A., *Symbol Digit Modalities Test (SDMT) Manual (Revised).* 1982, Western Psychological Services.: Los Angeles, CA.

4. Stroop, J.R., *Studies of interference in serial verbal reactions.*, 1935, Journal of Experimental Psychology.

5. Golden, C., *Stroop Color and Word Test: Cat. No. 30150M*, in *A Manual for Clinical and Experimental Uses*1978, Stoelting: Chicago, IL.

6. Reitan and R, *Validity of the trail making test as an indicator of organic brain damage*, 1958, Perceptual and Motor Skills. p. 271-276.

7. O'Rourke, J.J., et al., *The Trail Making Test in prodromal Huntington disease: contributions of disease progression to test performance.* J Clin Exp Neuropsychol, 2011. **33**(5): p. 567-79.

8. *Unified Huntington's Disease Rating Scale: reliability and consistency. Huntington Study Group.* Mov Disord, 1996. **11**(2): p. 136-42.

9. Paulsen, J.S., et al., *Clinical and Biomarker Changes in Premanifest Huntington Disease Show Trial Feasibility: A Decade of the PREDICT-HD Study.* Front Aging Neurosci, 2014. **6**: p. 78.

10. Consortium, G.M.o.H.s.D.G.-H., *Identification of Genetic Factors that Modify Clinical Onset of Huntington's Disease.* Cell, 2015. **162**(3): p. 516-26.
